# Surface Acting, Emotional Exhaustion, and Employee Sabotage to Customers: Moderating Roles of Quality of Social Exchanges

**DOI:** 10.3389/fpsyg.2018.02197

**Published:** 2018-11-14

**Authors:** Hui Zhang, Zhiqing E. Zhou, Yan Zhan, Chengbin Liu, Li Zhang

**Affiliations:** ^1^School of Sociology, Huazhong University of Science and Technology, Wuhan, China; ^2^Department of Psychology, Baruch College (CUNY), New York City, NY, United States; ^3^Department of Nursing, Taihe Hospital, Hubei University of Medicine, Shiyan, China; ^4^Cardiovascular Medicine Department Unit 3, Taihe Hospital, Hubei University of Medicine, Shiyan, China

**Keywords:** surface acting, emotional exhaustion, sabotage to customers, coworker exchange, leader-member exchange

## Abstract

Using the conservation of resources theory and social exchange theory as our conceptual frameworks, the current study examined how employee surface acting relates to their sabotage to customers through the mediating role of emotional exhaustion and explored the moderating roles of coworker exchange (CWX) and leader-member exchange (LMX). We collected two-wave time-lagged data from 540 clinical nurses and found that emotional exhaustion mediated the positive relationship between surface acting and employee sabotage to customers. In addition, we found that CWX buffered the positive effect of surface acting on emotional exhaustion, while LMX buffered the positive effect of emotional exhaustion on employee sabotage to customers, such that the effects were weaker when CWX and LMX were higher, respectively. These findings shed light on the effect of surface acting on employee harmful behaviors, the potential underlying mechanism, and boundary conditions to mitigate the negative consequences of surface acting.

## Introduction

Since the service industry accounts for above 60% of world GDP and the economy (The World Factbook, 2017), increasing research has focused on frontline service employees’ behaviors, attitudes, and feelings (Grandey, 2008; Mayer et al., 2009; Dong et al., 2015). During service delivery, however, employees have to conform to organizational expectations and goals to suppress negative emotions and display positive emotions, which is characterized as emotional labor (Ashforth and Humphrey, 1993; Brotheridge and Grandey, 2002). Emotional labor contains two different displaying rules: surface acting and deep acting. Surface acting emphasizes changing outward emotional display rather than altering the inner true feelings (Abraham, 1998; Grandey, 2000), whereas deep acting highlights regulating the inner feelings to meet requirements of the work (Grandey, 2000; Hülsheger and Schewe, 2011).

The current study examines the effect of surface acting on employee sabotage to customers and investigates potential underlying mechanisms and boundary conditions. We decided to only focus on surface acting for the following reasons. First, while many studies on emotional labor have investigated the outcomes of surface acting and deep acting in one study (e.g., Wagner et al., 2014; Deng et al., 2016), a large number of studies have only focused on surface acting (e.g., Prati et al., 2009; Shanock et al., 2013; Wang and Groth, 2014; Wagner et al., 2014; Krannitz et al., 2015). In addition, existing research on emotional labor suggests that deep acting has been linked with both positive and negative outcomes (Hülsheger and Schewe, 2011), while the literature consistently indicate that surface acting is negatively related to employee health, attitudes, performance, and well-being (Hülsheger and Schewe, 2011; Mesmer-Magnus et al., 2012), including increased emotional exhaustion (Grandey et al., 2012; Wagner et al., 2014; Yagil and Medlerliraz, 2017), higher negative affect (Judge et al., 2009), more strains (Hülsheger et al., 2010), lower job satisfaction (Giardini and Frese, 2006), lower work engagement (Bechtoldt et al., 2011), more work-to-family conflict (Wagner et al., 2014), higher turnover intentions (Becker et al., 2017), lower organizational commitment (Walsh et al., 2016), and lower task performance (Schmeichel et al., 2006; Goldberg and Grandey, 2007). Our study aims to build on this particular literature and expand the negative consequences of surface acting to employees sabotage to customers.

Second, recent literature indicates that surface acting may have an effect on employee harmful behaviors, such as interpersonal harmful behavior toward coworkers (Deng et al., 2016) and counterproductive behaviors at work (Bechtoldt et al., 2007). However, little is known about the potential effect of surface acting on employee harmful behaviors toward customers during the service interaction. As service employees have two roles at work, with one role being the member of the organization and one role providing service to customers (Côté et al., 2013), surface acting may not only affect their negative behaviors toward the organization and people working in the organization, but also their behaviors toward customers. Given that the accumulation of employee sabotage to customers may seriously decrease customer satisfaction (Schneider et al., 2005; Wang et al., 2011) and result in financial and reputational loss in service organizations (Harris and Ogbonna, 2002; Anderson et al., 2004), understanding whether and how surface acting predicts employee sabotage to customers will have potential implications to further understand how to reduce employee sabotage to customers.

Taken together, given the detrimental effects of surface acting and the focus on employee sabotage to customers as the outcome, in the current study we will examine the effect of employee surface acting on their sabotage to customers, and addresses why and how this might happen. We aim to make three contributions in the process. First, we contribute to the emotional labor literature by identifying employee sabotage to customers as an important negative consequence of surface acting. This illustrates that surface acting may have an impact on employee negative behaviors beyond coworkers and extend to customers during the interaction with customers, highlighting the importance of surface acting in the service industry.

Second, we draw on the conservation of resources theory (COR; Hobfoll, 1989) to theorize emotional exhaustion as the potential mechanism to understand why surface acting might lead to employee sabotage to customers. Surface acting can deplete employee resources and lead to emotional exhaustion (Judge et al., 2009; Grandey et al., 2012), and resource depletion can make employees less able to inhibit the impulse to engage in harmful behaviors under stress (Stucke and Baumeister, 2010), such as sabotage to customers. By identifying emotional exhaustion as the mediator, we will have a better understanding of the process from surface acting to sabotage to customers for theoretical implications, and potential ways to mitigate this process for practical implications.

Third, we examined social exchanges as moderators to buffer the negative effects of surface acting by supplying resources employees need. Previous studies have suggested that social exchanges in the workplace are important resources (Cohen and Wills, 1985; Ng and Sorensen, 2008; McCarthy et al., 2016; Schneider et al., 2017). Thus, we integrate the COR theory (Hobfoll, 1989) and Social Exchange Theory (Blau, 1964), and predict that coworker exchange (CWX) and leader-member exchange (LMX) may potentially buffer the effect of surface acting on employee sabotage to customers. It also emphasizes the importance of providing social recourses at work (McCarthy et al., 2016) to address interpersonal issues.

## Hypotheses Development

### Surface Acting and Sabotage to Customers

Surface acting has been considered a unique feature of service employees as part of their job (Cho et al., 2013). It refers to the requirement of front-line service employees to suppress the expression of their true feelings and express the false feelings during the interaction with customers (Gross, 1998; Grandey, 2000). As surface acting takes place during the interaction between employees and customers, it may not only have a harmful effect on employee behaviors toward coworkers (Deng et al., 2016) but also have an impact directly on customers during the interaction with customers, such as sabotage to customers. Sabotage to customers is common in the service industry as one type of interpersonal harming behavior (Crino, 1994; Skarlicki et al., 2008). It severely violates the service rule of providing friendly and professional high-quality service (e.g., Solomon et al., 1985; Skarlicki et al., 2008). Thus, understanding whether and why surface acting promotes employees engage in such behaviors will have important practical implications.

We propose that surface acting would positively predict employee sabotage to customers for the following reasons. First, displaying surface acting consumes employees’ resources to inhabit their true inner feelings, leading to resources loss or ego depletion (Judge et al., 2009; Wagner et al., 2014; Deng et al., 2016). Previous studies on ego depletion suggest that individuals will be more aggressive (Stucke and Baumeister, 2010), act unethically (Welsh et al., 2014), and behave in a more antisocial manner (Friehe and Schildberg-Hörisch, 2017) under resources depletion. Thus, when employees displaying surface acting and are depleted, they may have fewer resources to obey the organizational rules, leading to rule-breaking behaviors such as sabotage to customers.

Second, as surface acting requires employees to suppress their inner negative feelings, they are less likely to regulate their negative emotions and often are more likely to experience emotional dissonance (Deng et al., 2016). While employees might engage in organization deviance and harmful behaviors toward coworkers as a result (Bechtoldt et al., 2007; Deng et al., 2016), their actions might not stop there. Because of the frequent direct interaction between customers and front-line service employees, employees are also likely to engage in aggressive behaviors toward customers (Wang et al., 2011; Groth and Grandey, 2012). For example, when employees experience negative feelings from customers, they may allow themselves to put customers on hold for a longer period (Grandey, 2003; Wang et al., 2011; Groth and Grandey, 2012). These behaviors might be engaged to compensate for the suppressed negative feelings during the frequent interaction with customers.

In line with this, previous studies have found that the surface acting is positively associated with employee harmful interpersonal behaviors toward organization (Bechtoldt et al., 2007) and coworkers (Deng et al., 2016), which provide indirect empirical evidence of the possible impact of surface acting on employee sabotage to customers. In addition, previous research has found that customer-related experiences such as customer mistreatment (Wang et al., 2011; Groth and Grandey, 2012) and customer injustice (Skarlicki et al., 2016) can positively predict employee sabotage to customers. Given that employees tend to engage in surface acting as the initial reaction to customer-related negative experiences (Grandey et al., 2012), they are likely to engage in sabotage to customers when surface acting depletes too many resources that they are unable to inhibit their negative behaviors toward customers. Thus, we expect that surface acting has the potential positive effect on employee sabotage to customers and hypothesize that:

Hypothesis 1: Surface acting will positively predict employee sabotage to customers.

### Mediating Effect of Emotional Exhaustion

Emotional exhaustion is defined as a resource depletion state when a person no longer can make a big physical or mental effort (Gaines and Jermier, 1983). We employ the COR theory (Hobfoll, 1989) to explain the potential mechanism of the association between surface acting and employee sabotage to customers through emotional exhaustion. The COR theory suggests that individuals tend to protect and build resources important to them (e.g., energy and time; Hobfoll, 1989). Front-line employees often experience emotional exhaustion because they have to face excessive customer demands, resulting in resources depletion (Wright and Cropanzano, 1998). When the resources are depleted, they may experience a higher level of emotional exhaustion (Maslach et al., 2001). The COR theory also suggests that resources deplete over time, which is a long-term process (Hobfoll, 1989), and that it is faster for resources to deplete in coping with work demands than to supplement themselves (Freedy and Hobfoll, 1994). Surface acting requires expressubg inconsistent emotions with their inner feelings, and employees will have to devote more efforts to inhibiting impulse (Ashforth and Humphrey, 1993), consuming their resources and leading to emotional exhaustion. Previous research (e.g., Judge et al., 2009; Grandey et al., 2012; Wagner et al., 2014; Li et al., 2017; Kong and Jeon, 2018) has provided sufficient empirical evidence for this link, and it is expected the same in the current study.

The resource perspective (Hobfoll, 1989) can also provide an explanation of the relationship between emotional exhaustion and employee sabotage to customers. It has been suggested that depletion of control resources is an important reason for employees to engage in more deviant behaviors (Marcus and Schuler, 2004; Thau and Mitchell, 2010) and aggressive acts (Stucke and Baumeister, 2010), and thus emotional exhaustion, as the state of resource depletion, is likely to also predict employee sabotage to customers. When employees deplete their resources due to surface acting and experience emotional exhaustion, they are more likely to engage in inappropriate or undesirable behaviors because employees will have fewer resources to regulate these behaviors (e.g., Muraven et al., 1998). In addition, when employees are depleted with resources and experience emotional exhaustion, they also tend to ignore organizational rules or/and moral standards, result in rule-breaking and normative behaviors (Thau and Mitchell, 2010), such as sabotage to customers. Given the frequent direct interaction between customers and front-line service employees, when employees are experiencing high emotional exhaustion due to surface acting, they are less likely to inhibit impulsive behaviors and customers may become the available victims.

Although theoretically reasonable, the relationship between emotional exhaustion and employee sabotage to customers has not been empirically tested in previous studies. However, recent studies found that emotional exhaustion is positively associated with interpersonal harming behavior, such as interpersonal harming to coworkers and aggression in organization (e.g., Thau and Mitchell, 2010; Christian and Ellis, 2011; Wang et al., 2011; Deng et al., 2016), which suggest that employees with a higher level of emotional exhaustion may also engage in interpersonal harming behavior toward customers such as sabotage to customers.

Hypothesis 2: Emotional exhaustion will positively predict employee sabotage to customers.

According to the COR theory (Hobfoll, 1989) as well as the theoretical argument and empirical evidence presented above, we believe surface acting can consume employees’ resources and result in emotional exhaustion, which in turn will lead to employee sabotage to customers. Thus we argue that emotional exhaustion links surface acting and employee sabotage to customers and predict that:

Hypothesis 3: Employees’ emotional exhaustion mediates the relationship between surface acting and sabotage to customers.

### Moderating Effects of Social Exchanges

The COR theory (Hobfoll, 1989) also highlights the conditions to protect individuals form resources losses and to cope with resources losses. It suggests that when facing potential or actual resources losses, individuals often tend to gain available resources to supply and protect resources (Hobfoll, 1989). Social support is one of the important ways in this process (Hobfoll, 1989, 2001). First, social support can provide resources to broaden individuals’ resources pool (Hobfoll, 1989) and help people alleviate the negative effect caused by resource-depleting experiences (e.g., surface acting) through a few channels, including promoting skills to cope with demands (Dunahoo et al., 1998) and decreasing work demands (Ray and Miller, 1994) and emotional dissonance (Monica et al., 2016). Second, social support also promotes the replenishment of resources pool and formation of the gain spirals after resource loss (Hobfoll, 1989) and thus buffers the negative effects of resource loss state such as emotional exhaustion (Hakanen et al., 2008).

According to the social exchange theory, there are two types of important social support as resources supplement in the workplace: leader-member exchange (LMX, support from leaders; Graen and Uhl-Bien, 1995) and coworker exchange (CWX, support from coworkers; Sherony and Green, 2002). Employees can gain social support resources form the interaction with coworkers and leaders in the work context to cope with work demands (Ng and Sorensen, 2008; Monica et al., 2016) and resource depletion (McCarthy et al., 2016). Therefore, we propose that employees receiving more social support from coworkers and leaders through high levels of CWX and LMX, respectively, will gain and supply resources and mitigate the negative effects of surface acting and emotional exhaustion caused by surface acting.

### The Moderating Role of CWX

Although CWX and LMX are both important resources for employees, their roles might be different. Compared to the relationship between leader and employees, the relationship between coworkers is more equal and less performance monitoring (Diefendorff and Greguras, 2009), and focusing more on trust and social reciprocity (Cole et al., 2002). The social exchange theory (Blau, 1964) suggests that more authentic, intimate and personal social exchange is based on reciprocity and social resources, while more economic and transactional exchanges are based on materialistic and instrument resources. Thus, employees may gain more social and emotional resources from the social exchange with coworkers (CWX), but gain more instrument resources from the economic exchange with leaders (LMX; McCarthy et al., 2016).

As coworkers own equal power and interpersonal relationship with employees, employees are more likely to share emotional events at work with coworkers (Hadley, 2014). The more frequent interaction between employees and coworkers than leaders provides social support with behavioral and emotional resources (Chiaburu and Harrison, 2008) and employees can receive more social resources to cope work demands and strains (Thoits, 2011). In line with this notion, while surface acting as a typical work strains for front-line service employees can deplete their emotional resources and result in emotional exhaustion, CWX can provide emotional resources (Karasek et al., 1982; Wu and Hu, 2009) to buffer the positive effect of surface acting on emotional exhaustion.

Although previous studies have not investigated the mitigating effect of CWX in the association between surface acting and emotional exhaustion, research on coworker support provides indirect empirical evidence of the moderating effect. For example, coworker support moderates the effects of abusive supervision on emotional exhaustion (Wu and Hu, 2009), and the relationship between workplace anxiety and emotional exhaustion (McCarthy et al., 2016). In addition, a meta-analysis by Viswesvaran et al. (1999) found that social support from coworkers has a stronger mitigating effect on the stressor-strain relation than social support from leaders. Because surface acting serves as a stress source for service employee, the similar buffering effect of CWX on the effect of surface acting on emotional exhaustion can be expected. Combining the theoretical argument and empirical evidence, we propose that CWX will moderate the relationship between surface acting and emotional exhaustion.

Hypothesis 4: CWX will moderate the relationship between surface acting and emotional exhaustion, such that the positive relationship will be weaker when CWX is high.

### Moderating Role of LMX

We also predict that LMX as support from leaders may buffer the effect of emotional exhaustion on employee sabotage to customers. As leaders have more power and high state than employees (Diefendorff et al., 2010), the interaction between leaders and employees involves less emotional sharing but more economic exchange (Hüffmeier and Hertel, 2011). Because high LMX provides important material and instructional resources for employees to supply their resource pool, they are likely to use such instrument resources to overcome emotional exhaustion and regulate their behaviors (Ng and Sorensen, 2008; McCarthy et al., 2016) and perform better (Dulebohn et al., 2012; Jiang et al., 2014).

Further, according to the dual level social exchange theory (Schaufeli et al., 1996), employees are more likely to build a balanced reciprocity relationship with leaders and organizations. Based on the economic exchange, a higher level of LMX may lead to more employees positive behaviors benefiting the company and fewer negative behaviors. Thus employees receiving more support from leaders are more likely to feel the obligation to engage in positive behaviors (Sakurai and Jex, 2012) and perform effectively (McCarthy et al., 2016), and are less likely to engage in sabotage to customers even when experiencing emotional exhaustion due to resource losses.

Previous studies on LMX also provide indirect empirical evidence of the moderating effect. LMX buffers the relationship between emotional exhaustion and performance (McCarthy et al., 2016), and supervisor social support buffers the effect of negative emotions and both work effort on CWBs (Sakurai and Jex, 2012). Thus, we propose that:

Hypothesis 5: LMX will moderate the relationship between emotional exhaustion and sabotage to customers, such that the positive relationship will be weaker when LMX is high.

**FIGURE 1:**
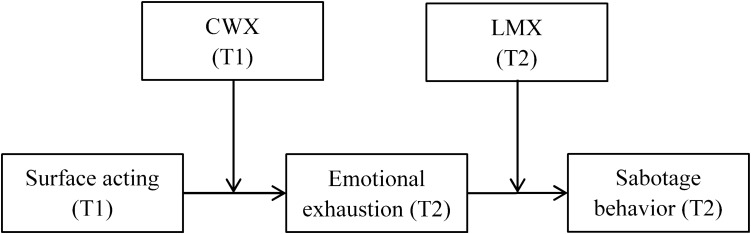
Theoretical model.

Figure 1 summarizes the relationships proposed in the hypotheses above.

## Materials and Methods

### Participants and Procedure

Emotion labor has been mainly studied with samples from service industries such as hotels, hospitals, restaurants, airline services, call centers and transit companies. Nurses in hospitals interact with patients and their family members in their daily work, and they are expected to provide good customer service to patients (Drach-Zahavy, 2010), often requiring emotional labor (Ashforth and Humphrey, 1993). Pervious studies also suggested that nurses in hospitals experience a high level of surface acting at work (Grandey et al., 2012). Thus, we decided to use nurses as our sample for the current study. We collected data from seven large hospitals in China across two waves. We first obtained permission from the hospitals’ directors and their ethical committees to conduct the study. We then met with head nurses from each department to explain the aims and requirements of the current study, and we asked them to encourage nurses to participate in the survey. After that, we sent out 800 questionnaires supervisors of departments who helped pass the questionnaires onto their followers. Each participant was told that they would receive 10 China Yuan ($1.52) as compensation for their time. Two days later, we returned to the hospitals to collect the surveys and received 640 questionnaires, with a response rate of 80.00%. The first survey included measures of demographic variables, surface acting, and CWX.

Three months after time 1, all employees who completed Time 1 survey received a second questionnaire that assessed their emotional exhaustion, LXM, and sabotage to customers. Participants received a box of chocolate as an incentive gift for returning the survey. A total of 540 Time 2 surveys could be matched with a corresponding survey at time 1, of whom 95.20% were female (*n* = 540). The average age was 30.17 years (*SD* = 5.51) and their average tenure in their hospital was 8.01 years (*SD* = 6.35).

### Measures

Chinese versions of the followings measures were administered to participants in the current study. We used the translation and back-translation method (Brislin, 1980) to translate the scales from English into Chinese to make an equivalent meaning. Further, as the participants were clinic nurses in hospitals, we followed the suggestion of Schaffer and Riordan (2003) to modify some wording to ensure the applicability of the hospital context.

#### Surface Acting

A five-item of emotions labor scale developed by Brotheridge and Lee (2002) was used to measure surface acting. Participants rated items on a five-point frequency scale from 1 = *never* to 5 = always. An example item is “Put on an act in order to display for my job” (α = 0.82).

#### Coworker Exchange

We used a seven-item scale originally developed by Graen and Uhl-Bien (1995) and later modified by McCarthy et al. (2016) to measure CWX by replacing “supervisor” with “coworkers.” An example item is “My coworkers understand my job problems and needs” (α = 0.97).

#### Emotional Exhaustion

We used a five-item subscale of the Maslach Burnout Inventory General Survey developed by Schaufeli et al. (1996) to measure emotional exhaustion. Participants rated items on a seven-point Likert scale from 1 (*never*) to 7 (every day). An example item is “I feel tired after get up when I have to deal with work in the day” (α = 0.96).

#### Leader-Member Exchange

Leader-member exchange was assessed with a seven-item scale developed by Graen and Uhl-Bien (1995). Participants rated the items about their supervisor from 1 (*strongly disagree*) to 5 (*strongly agree*). An example item is “My supervisor understand my job problems and needs” (α = 0.97).

#### Sabotage to Customers

We used Skarlicki et al. (2008) five-item scale to measure sabotage to customers. To fit the context for the nurses, we modified the words “customers” in the scale into “patients” in the current study. Participants rated items on a five-point Likert scale from 1 = never to 5 = frequently. An example item is “Hung up on the patients” (α = 0.84).

#### Control Variables

We controlled for nurses’ demographic variables (i.e., age, job tenure, and education) on the study variables (i.e., surface act, emotional exhaustion, and sabotage to customers; Dahling and Perez, 2010; Wang et al., 2011). As deep acting is associated with ego depletion (Deng et al., 2016) and resources replenishment (Hülsheger and Schewe, 2011), we controlled deep acting when examining the relationships of surface acting with the outcome variables. Deep acting was measured with a three-item of emotion labor scale developed by Brotheridge and Lee (2002). Participants rated items on a five-point frequency scale from 1 = never to 5 = always. An example item is “Work hard to feel the emotions that I need to show to others” (α = 0.81).

## Results

### Confirmatory Factor Analysis

A confirmatory factor analysis was conducted via Amos 21.0 to establish discriminant validity of the study variables. As shown in Table 1, the hypothesized five-factor model provides a better fit to the data [χ^2^ (*df* = 367) = 1062.29; RMSEA = 0.06, TLI = 0.95, CFI = 0.95] than all alternative models, showing evidence of discriminant validity.

**Table 1:** Results of confirmatory factor analysis.

Model	χ^2^	*df*	χ^2^/*df*	CFI	TLI	RMSEA
Hypothesized five-factor model	1062.29	367	2.89	0.95	0.95	0.06
Three-factor model (emotional exhaustion, CWX, LMX combined into one factor)	8992.64	374	24.05	0.43	0.39	0.21
One-factor model (All five factors were combined into one factor)	10889.13	377	28.88	0.31	0.26	0.23

*N = 540. CFI, comparative fit index; TLI, Tucker–Lewis index; RMSEA, root mean square error of approximation. CWX, coworker exchange; LMX, leader-member exchange.*

### Descriptive Statistics

Table 2 shows means, standard deviations, and correlations of the study variables. Consistent with our hypotheses, the correlations among surface acting, emotional exhaustion, and sabotage behavior to customers were all in the expected direction. This suggested that it was appropriate to conduct formal mediation analyses to test our hypotheses. Because of the high correlations between age and job tenure (*r* = 0.95, *p* < 0.001), we only control job tenure in the following analysis.

**Table 2:** Means, standard deviations, and correlations among the study variables.

Variable	*M*	*SD*	1	2	3	4	5	6	7	8	9
(1) Age	30.17	5.51	–								
(2) Job tenure	8.01	6.35	0.95^∗∗∗^	–							
(3) Education	–	–	−0.19^∗∗^	−0.22^∗∗^	–						
(4) Deep acting	3.91	0.64	0.08	0.07	−0.03	–					
(5) Surface acting	3.32	0.74	0.11^∗∗^	0.09^∗^	−0.03	0.24^∗∗^	–				
(6) Emotional exhaustion	3.28	1.39	0.004	−0.002	0.01	0.03	0.41^∗∗^	–			
(7) CWX	2.55	1.13	−0.003	−0.02	−0.01	−0.07	0.10^∗^	−0.06	–		
(8) LMX	2.59	1.11	−0.10^∗^	−0.09^∗^	−0.02	−0.24^∗∗^	−0.03	0.04	0.04	–	
(9) Sabotage to customers	1.79	0.79	−0.04	0.09^∗^	0.09^∗^	−0.08	0.34^∗∗^	0.33^∗∗^	0.09^∗^	–0.04	–

*N = 540. ^∗^p < 0.05, ^∗∗^p < 0.01, ^∗∗∗^p < 0.001. CWX, coworker exchange; LMX, leader-member exchange.*

### Hypothesis Testing

We used the PROCESS macro for SPSS (Hayes, 2013) to test our hypotheses. As shown in Table 3, after controlling job tenure, education and deep acting in model 3, surface acting positively predicted employee sabotage to customers (*B* = 0.41, *p* < 0.001). Hence, Hypothesis 1 was supported. In support of Hypothesis 2, the result showed that emotional exhaustion positively predicted employee sabotage to customers in model 4 (*B* = 0.19, *p* < 0.001). After entering emotional exhaustion, surface acting was less significantly associated with employee sabotage to customers (*B* = 0.31, *p* < 0.001), whereas emotional exhaustion (*B* = 0.12, *p* < 0.001) was positively related to employee sabotage to customers in model 5. Furthermore, we calculated the indirect effect of surface acting on employee sabotage to customers through emotional exhaustion with 5,000 bootstrapped samples. The result showed that surface acting had a significant indirect effect on employee sabotage to customers through emotional exhaustion (indirect effect = 0.10, 95% CI [0.06,0.16]). Thus, Hypotheses 3 was supported.

**Table 3:** Results of regression analyses.

Variable	Emotional exhaustion		Sabotage to customers			
Model Intercept	Model 1	Model 2	Model 3	Model 4	Model 5	Model 6
	1.38^∗^ (0.54)	0.91 (0.51)	1.02^∗^ (0.31)	0.12^∗∗∗^ (0.31)	0.85^∗^ (0.34)	2.35^∗∗∗^ (0.30)
Job tenure	−0.01 (0.01)	−0.01 (0.01)	−0.01 (0.01)	−0.004 (0.01)	−0.01 (0.004)	−0.01 (0.005)
Education	−0.04 (0.12)	−0.04 (0.12)	0.10 (0.08)	0.13 (0.08)	0.11 (0.07)	0.10 (0.07)
Deep acting	−0.17 (0.09)	−0.18^∗^ (0.09)	−0.21^∗∗∗^ (0.05)	−0.11^∗^ (0.05)	−0.19^∗∗^ (0.05)	−0.20^∗∗^ (0.05)
Surface acting	0.82^∗∗∗^ (0.04)	0.81^∗∗∗^ (0.09)	0.41^∗∗∗^ (0.04)		0.31^∗∗∗^ (0.04)	0.29^∗∗∗^ (0.04)
Emotional exhaustion				0.19^∗∗∗^ (0.02)	0.12^∗∗∗^ (0.03)	0.12^∗∗∗^ (0.03)
CWX		−0.12^∗^ (0.05)				
LMX						−0.06^∗^ (0.03)
Surface acting^∗^CWX		−0.24^∗∗^ (0.12)				
Emotional exhaustion^∗^LMX						−0.09^∗∗∗^ (0.02)
*δR*^2^	0.18^∗∗∗^	0.20^∗∗∗^	0.14^∗∗∗^	0.12^∗∗∗^	0.19^∗∗∗^	0.23^∗∗∗^
*F*	25.72^∗∗∗^	28.11^∗∗∗^	23.45^∗∗∗^	18.97^∗∗∗^	19.59^∗∗∗^	14.79^∗∗∗^

*N = 540. ^∗^p < 0.05, ^∗∗^p < 0.01, ^∗∗∗^p < 0.001. CWX, coworker exchange; LMX, leader-member exchange.*

**FIGURE 2:**
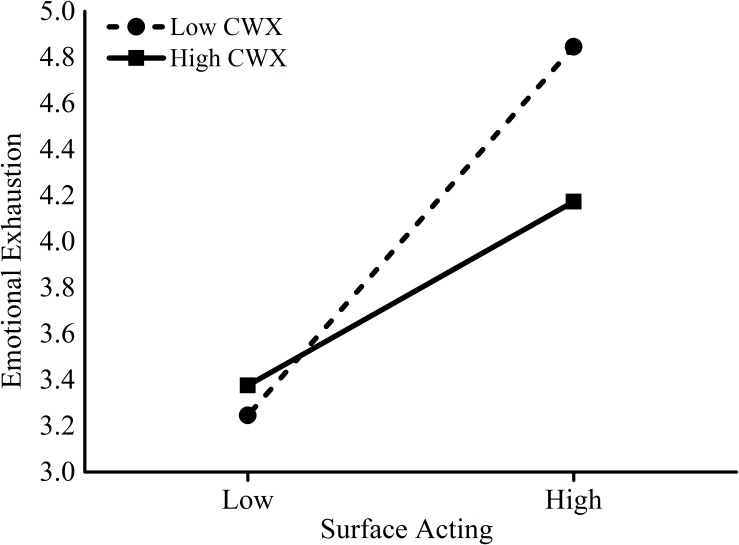
Coworker exchange (CWX) as a moderator of surface acting and emotional exhaustion.

**FIGURE 3:**
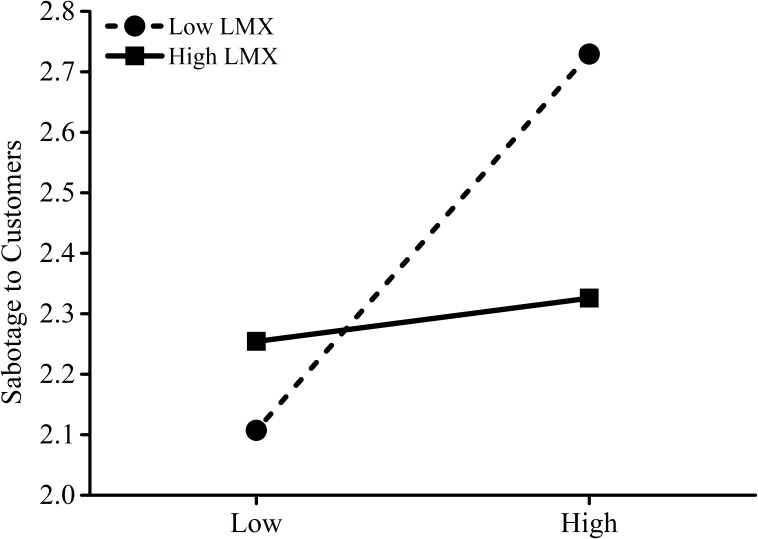
Leader-member exchange (LMX) as moderator of emotional exhaustion and sabotage behavior.

Hypothesis 4 and Hypothesis 5 focused on the moderating effects of CWX on the association between surface acting and emotional exhaustion, and LMX on the relationship between emotional exhaustion and employee sabotage behavior to customers, respectively. We added the interactions term between surface acting and CWX into model 2 in Table 3 to test Hypothesis 4. The interaction effect was significant (*B* = −0.24, *p* = 0.05). Figure 2 further revealed that when CWX was lower, the positive effect of surface acting on emotional exhaustion was stronger (*B* = 1.08, *t* = 10.07, *p* < 0.001) than when CWX was higher (*B* = 0.55, *t* = 2.70, *p* = 0.007). Thus, Hypothesis 4 was supported. We added the interactions term between LMX and emotional exhaustion into model 6 in Table 3 to test Hypothesis 5. The interaction effect was also significant (*B* = −0.09, *p* < 0.001). As shown in Figure 3, the positive effect of emotional exhaustion on employee sabotage behavior to customers was stronger (*B* = 0.22, *t* = 7.92, *p* < 0.001) when LMX was lower than when LMX was higher (*B* = 0.03, *t* = 0.51, *p* = 0.61), supporting Hypothesis 5.

## Discussion

Our study found that surface acting has a positive effect on employee sabotage to customers through emotional exhaustion. Further, social exchanges buffer the negative effects. Specifically, the positive relationship between surface acting and emotional exhaustion is weaker for individuals with high CWX, and the positive relationship between emotional exhaustion and employee sabotage to customers is weaker for individuals with high LMX. Our findings suggest that while surface acting is ubiquitous and may result in serious negative consequences in service industries, social support from coworkers and leaders can potentially alleviate the harmful effects.

### Theoretical Implications

Our findings offer important theoretical insights. First, we contribute to the emotional labor literature by extending the effect of surface acting to employees sabotage to customers. Previous studies have suggested surface acting is positively related to employee harmful behaviors within organizations, such as deviance toward the organization (Bechtoldt et al., 2007) and harmful behavior toward coworkers (Deng et al., 2016). Our study suggests that surface acting might also lead to employee harmful behaviors toward customers during the service delivery. Surface acting might consume employees’ resources to suppress their true inner feelings and result in resources depletion (Judge et al., 2009; Wagner et al., 2014; Deng et al., 2016), which might make employees be more antisocial and aggressive (Stucke and Baumeister, 2010; Friehe and Schildberg-Hörisch, 2017) during the interaction with customers, eliciting sabotage to customers. The findings supported the notion that surface acting has a broader impact and social cost not only within organizations (Deng et al., 2016), at home (Wagner et al., 2014), but also in service encounters. In addition, we contribute to the increasing literature on potential antecedents of employee sabotage to customers (e.g., Wang et al., 2011; Groth and Grandey, 2012; Skarlicki et al., 2016) and suggest that employee surface acting also has a potential to lead to their own harmful behaviors toward customers.

Second, based on the COR theory (Hobfoll, 1989), we contribute to the literature by finding that surface acting might promote employee sabotage to customers through emotional exhaustion. As previous studies (e.g., Judge et al., 2009; Grandey et al., 2012; Wagner et al., 2014; Li et al., 2017; Kong and Jeon, 2018) have found, surface acting positively predicted emotional exhaustion, suggesting that surface acting might require employees to devote resources to suppress emotion impulses (Ashforth and Humphrey, 1993). Further, when people experience emotional exhaustion as a state of depletion of control resources, they are more likely to engage deviant behaviors (Marcus and Schuler, 2004; Thau and Mitchell, 2010) and aggressive acts (Stucke and Baumeister, 2010), which might also extend to customers and lead to sabotage to customers. Our finding on the mediating effect of emotional exhaustion in the relationship between surface acting and employee sabotage to customers is consistent with this argument.

Third, based on the COR theory, our results show that social support from coworkers and leader can buffer the harmful effect of surface acting. CWX and LMX, as the main sources of social support in the workplace (McCarthy et al., 2016), have been found useful in providing resources to buffer the negative effect of work demands (Ng and Sorensen, 2008; Monica et al., 2016), such as surface acting and resource depletion (McCarthy et al., 2016). Our findings further demonstrate the importance of CWX and LMX in the resource depletion process of surface acting. On one hand, CWX can provide emotional resources (Karasek et al., 1982; Wu and Hu, 2009) to buffer the positive effect of surface acting on emotional exhaustion; on the other hand, LMX can provide more instrument resources to overcome emotional exhaustion and regulate their behaviors (Ng and Sorensen, 2008; McCarthy et al., 2016) to reduce employee sabotage to customers. This finding also adds to the previous literature that have demonstrated that individual factors such as emotion regulation self-efficacy (Deng et al., 2016) and situatonal factor such as climate of authenticity (Grandey et al., 2012; Li et al., 2017) can moderate the relationship between surface acting and resource losses. Our findings show that interpersonal factors such as CWX and LMX can be potential resource supplements to buffer the positive effect of surface acting and emotional exhaustion.

### Practical Implications

Our study provides important practical implications for organizations where service employees display surface acting frequently. First, surface acting might seriously harm the organizations when employees engage in sabotage to customers as a response. Service organizations should pay more attention to employees’ emotions and train them to better deal with the negative emotions caused by customers. Previous research has suggested that perspective taking is an important way to decrease the employees’ negative affect (Parker and Axtell, 2001; Bechtoldt et al., 2007), so that employees may not make efforts to regulate emotions or suppress negative affect to reduce the frequency of displaying surface acting. Besides, Li et al. (2017) found that employee mindfulness is negative associated with surface acting. Thus, service organizations can train their employees to master the strategy of perspective taking and be mindfulness. In addition, organizations can also train employees to engage in more deep acting (Deng et al., 2016) to reduce the occurrence of surface acting. Furthermore, organizations should also try to reduce internal sources of surface acting when it is inevitable from customers. For example, abusive supervision (Chi et al., 2018) and coworker interpersonal mistreatment (Adams and Webster, 2013) have been found to positively relate to surface acting, and organizations should try to establish a more supportive climate to reduce these experiences of employee and subsequent surface acting.

Second, given that emotional exhaustion might mediate the relationship between surface acting and sabotage to customers, it is vital for service employees to gain resources to recover from emotional exhaustion, which may in turn reduce sabotage to customers. Sonnentag and Fritz (2007) suggested that effective recovery activities including relaxation, psychological detachment, exerting personal control, and engaging in mastery experiences can be potential ways to recovery from emotional exhaustion. Besides, service organizations should also supply more opportunities for employees to reduce emotional demands (Goldberg and Grandey, 2007), have a rest (Trougakos et al., 2008), or engage in more social sharing (Baranik et al., 2017). Further, some targeted interventions on ego depletion also should take into consideration (Awa et al., 2010).

Third, the current study demonstrates the buffering effects of social exchange in both stages of the relationship between surface acting and sabotage to customers through emotional exhaustion. Thus, service organizations should try to promote high-quality relationships among employees, and between employees and their leaders. For example, Miles et al. (1996) suggest that open communication is a significant strategy to develop these relations. Besides, from the perspective of leadership, previous studies have suggested that the positive leadership, such as servant leadership, plays a key role in developing LMX and support climate (Wu et al., 2013; Liden et al., 2015). Thus organizations should encourage supervisors to serve as servant leaders and pay more attention to employee development and give priority to their needs and interests.

### Limitations and Future Directions

Our study has a few limitations. First, although we collected two waves of data to reduce common method variance, measuring all the variables using the same source can still raise potential concerns about common method variance. Future studies may use a third party to observe and report about the service interactions, such as coworkers perceived CWX, leaders perceived LMX, and customer reported sabotage to customers, which might provide a more objective assessment of employee behaviors and interactions. In addition, we can’t draw conclusions concerning causality. Thus, future research may attempt to conduct longitudinal or experimental designs manipulating surface acting to verify causality.

Second, the data used in our studies were collected from nurses in China, limiting the generalizability of our findings; future studies should further replicate our findings with samples from other service industries such as hotels, banks, and airlines and other regions to extend our understanding of the effect of surface acting on employee harmful behaviors toward customers.

Third, our findings indicated that emotional exhaustion mediated the relationship between surface acting and employees sabotage to customers. However, it is likely that other mechanisms also exist. For example, integrating insights from work meaning theory (Rosso et al., 2010) may advance our understanding of how surface acting might result in more sabotage to customers through decreased work meaning. Besides, we only examined social exchange as potential moderators, and future research should further examine some other contextual factors such as climate of authenticity (Li et al., 2017) and individual factors such as emotion regulation self-efficacy (Deng et al., 2016) and emotional intelligence (Prati et al., 2009) as potential buffers.

## Conclusion

Drawing upon the COR theory (Hobfoll, 1989) and social exchange theory (Blau, 1964), our study provides support for the mediating effect of emotional exhaustion in the relationship between surface acting and employee sabotage to customers, and social exchange as boundary conditions to buffer the effect of surface acting on employee emotional exhaustion and the effect of emotional exhaustion on sabotage to customers. These findings shed light on employee harmful behaviors as potential consequences of surface acting, as well as the potential underlying mechnisms and boundary couditions.

## Ethics Statement

An ethics approval was not required as perinstitutional guidelines and national laws and regulations because no unethical behaviors existed in this study. We just conducted paper–pencil test and were exempt from further ethics board approval since this research did not involve human clinical trials or animal experiments. All subjects gave written informed consent in accordance with the Declaration of Helsinki. Research respondents were ensured confidentiality and anonymity. All participation was voluntary.

## Author Contributions

HZ, ZZ, YZ, and CL: making substantial contributions to design, models, and hypotheses. HZ, YZ, and LZ: acquisition of data. HZ, ZZ, and YZ: analysis and interpretation of data. HZ, ZZ, and CL: wrote and revised the article. HZ, ZZ, YZ, CL, and LZ: final approval.

## Conflict of Interest Statement

The authors declare that the research was conducted in the absence of any commercial or financial relationships that could be construed as a potential conflict of interest.

## References

[B1] Abraham R. (1998). Emotional dissonance in organizations: antecedents, consequences, and moderators. *Genet. Soc. Gen. Psychol. Monogr.* 124 229–246. 9597747

[B2] Adams A. G. Webster R. J. (2013). Emotional regulation as a mediator between interpersonal mistreatment and distress. *Eur. J. Work Organ. Psychol.* 22 697–710. 10.1080/1359432X.2012.698057

[B3] Anderson E. W. Fornell C. Mazvancheryl S. K. (2004). Customer satisfaction and shareholder value. *J. Mark.* 68 172–185. 10.1509/jmkg.68.4.172.42723

[B4] Ashforth B. E. Humphrey R. H. (1993). Emotional labor in service roles: the influence of identity. *Acad. Manag. Rev.* 18 88–115. 10.5465/AMR.1993.3997508 23216225

[B5] Awa W. L. Plaumann M. Walter U. (2010). Burnout prevention: a review of intervention programs. *Patient Educ. Couns.* 78 184–190. 10.1016/j.pec.2009.04.008 19467822

[B6] Baranik L. E. Wang M. Gong Y. Shi J. (2017). Customer mistreatment, employee health, and job performance: cognitive rumination and social sharing as mediating mechanisms. *Soc. Sci. Electron. Publishing* 29 1971–1977. 10.1177/0149206314550995

[B7] Bechtoldt M. N. Rohrmann S. De Pater I. E. Beersma B. (2011). The primacy of perceiving: emotion recognition buffers negative effects of emotional labor. *J. Appl. Psychol.* 96 1087–1094. 10.1037/a0023683 21574676

[B8] Bechtoldt M. N. Welk C. Hartig J. Zapf D. (2007). Main and moderating effects of self-control, organizational justice, and emotional labor on counterproductive behavior at work. *Eur. J. Work Organ. Psychol.* 16 479–500. 10.1080/13594320701662618

[B9] Becker W. J. Cropanzano R. Wagoner P. V. Keplinger K. (2017). Emotional labor within teams: outcomes of individual and peer emotional labor on perceived team support, extra-role behaviors, and turnover intentions. *Group Organ. Manag.* 43 38–71. 10.1177/1059601117707608

[B10] Blau P. M. (1964). Justice in social exchange. *Sociol. Inq.* 34 193–206. 10.1111/j.1475-682X.1964.tb00583.x

[B11] Brislin R. W. (1980). “Translation and content analysis of oral and written materials,” in *Handbook of Cross-Cultural Psychology*, eds Triandis H. C. Berry J. W. (Boston, MA: Allyn & Bacon), 389–444.

[B12] Brotheridge C. M. Grandey A. A. (2002). Emotional labor and burnout: comparing two perspectives of “people work”. *J. Vocat. Behav.* 60 17–39. 10.1006/jvbe.2001.1815

[B13] Brotheridge C. M. Lee R. T. (2002). Testing a conservation of resources model of the dynamics of emotional labor. *J. Occup. Health Psychol.* 7 57–67. 10.1037/1076-8998.7.1.57 11827234

[B14] Chi N. W. Chen Y. C. Huang T. C. Chen S. F. (2018). Trickle-down effects of positive and negative supervisor behaviors on service performance: the roles of employee emotional labor and perceived supervisor power. *Hum. Perform.* 31 55–75. 10.1080/08959285.2018.1442470

[B15] Chiaburu D. S. Harrison D. A. (2008). Do peers make the place? conceptual synthesis and meta-analysis of coworker effects on perceptions, attitudes, ocBs, and performance. *J. Appl. Psychol.* 93 1082–1103. 10.1037/0021-9010.93.5.1082 18808227

[B16] Cho Y. N. Rutherford B. N. Park J. K. (2013). Emotional labor’s impact in a retail environment. *J. Bus. Res.* 66 2338–2345. 10.1016/j.jbusres.2012.04.015

[B17] Christian M. S. Ellis A. P. J. (2011). Examining the effects of sleep deprivation on workplace deviance: a self-regulatory perspective. *Acad. Manag. J.* 54 913–934. 10.5465/amj.2010.0179

[B18] Cohen S. Wills T. A. (1985). Stress, social support, and the buffering hypothesis. *Psychol. Bull.* 98 310–357. 10.1037/0033-2909.98.2.310 3901065

[B19] Cole M. S. Schaninger W. S. Harris S. G. (2002). The workplace social exchange network: a multilevel, conceptual examination. *Group Organ. Manag.* 27 142–167. 10.1177/1059601102027001008

[B20] Côté S. Van Kleef G. A. Sy T. (2013). “The social effects of emotion regulation in organizations,” in *Emotional Labor in the 21st Century: Diverse Perspectives on Emotion Regulation at Work*, eds Grandey A. A. Diefendorff J. M. Rupp D. E. (New York, NY: Psychology Press/Routledge), 79–100.

[B21] Crino M. D. (1994). Employee sabotage: a random or preventable phenomenon? *J. Manag. Issues* 6 311–330.

[B22] Dahling J. J. Perez L. A. (2010). Older worker, different actor? linking age and emotional labor strategies. *Pers. Individ. Diff.* 48 574–578. 10.1016/j.paid.2009.12.009

[B23] Deng H. Walter F. Lam C. K. Zhao H. H. (2016). Spillover effects of emotional labor in customer service encounters toward coworker harming: a resource depletion perspective. *Pers. Psychol.* 70 469–502. 10.1111/peps.12156

[B24] Diefendorff J. Morehart J. Gabriel A. (2010). The influence of power and solidarity on emotional display rules at work. *Motiv. Emot.* 34 120–132. 10.1007/s11031-010-9167-8

[B25] Diefendorff J. M. Greguras G. J. (2009). Contextualizing emotional display rules: examining the roles of targets and discrete emotions in shaping display rule perceptions. *J. Manag.* 35 880–898. 10.1177/0149206308321548

[B26] Dong Y. Liao H. Chuang A. Zhou J. Campbell E. M. (2015). Fostering employee service creativity: Joint effects of customer empowering behaviors and supervisory empowering leadership. *J. Appl. Psychol.* 100 1364–1380. 10.1037/a0038969 25774571

[B27] Drach-Zahavy A. (2010). How does service workers’ behavior affect their health? service climate as a moderator in the service behavior–health relationships. *J. Occup. Health Psychol.* 15 105–119. 10.1037/a0018573 20364909

[B28] Dulebohn J. H. Bommer W. H. Liden R. C. Brouer R. L. Ferris G. R. (2012). A meta-analysis of antecedents and consequences of leader-member exchange: integrating the past with an eye toward the future. *J. Manag.* 38 1715–1759. 10.1177/0149206311415280

[B29] Dunahoo C. L. Hobfoll S. E. Monnier J. Hulsizer M. R. Johnson R. (1998). There’s more than rugged individualism in coping. part 1: even the lone ranger had tonto. *Anxiety Stress Coping* 11 137–165. 10.1080/10615809808248309

[B30] Freedy J. R. Hobfoll S. E. (1994). Stress inoculation for reduction of burnout: a conservation of resources approach. *Anxiety Stress Coping* 6 311–325. 10.1080/10615809408248805

[B31] Friehe T. Schildberg-Hörisch H. (2017). Predicting norm enforcement: the individual and joint predictive power of economic preferences, personality, and self-control. *Eur. J. Law Econ.* 45 1–20. 10.1007/s10657-017-9556-5

[B32] Gaines J. Jermier J. M. (1983). Emotional exhaustion in a high stress organization. *Acad. Manag. J.* 26 567–586. 10.2307/255907

[B33] Giardini A. Frese M. (2006). Reducing the negative effects of emotion work in service occupations: emotional competence as a psychological resource. *J. Occup. Health Psychol.* 11 63–75. 10.1037/1076-8998.11.1.63 16551175

[B34] Goldberg L. S. Grandey A. A. (2007). Display rules versus display autonomy: emotion regulation, emotional exhaustion, and task performance in a call center simulation. *J. Occup. Health Psychol.* 12 301–318. 10.1037/1076-8998.12.3.301 17638495

[B35] Graen G. B. Uhl-Bien M. (1995). Relationship-based approach to leadership: development of leader-member exchange (lmx) theory of leadership over 25 years: applying a multi-level multi-domain perspective. *Leadersh. Q.* 6 219–247. 10.1016/1048-9843(95)90036-5

[B36] Grandey A. Foo S. C. Groth M. Goodwin R. E. (2012). Free to be you and me: a climate of authenticity alleviates burnout from emotional labor. *J. Occup. Health Psychol.* 17 1–14. 10.1037/a0025102 21875210

[B37] Grandey A. A. (2000). Emotion regulation in the workplace: a new way to conceptualize emotional labor. *J. Occup. Health Psychol.* 5 95–110. 10.1037/1076-8998.5.1.95 10658889

[B38] Grandey A. A. (2003). When “the show must go on”: surface acting and deep acting as determinants of emotional exhaustion and peer-rated service delivery. *Acad. Manag. J.* 46 86–96. 10.2307/30040678

[B39] Grandey A. A. (2008). “Emotions at work: a review and research agenda,” in *Handbook of Organizational Behavior*, eds Cooper C. Barling J. (London: Sage), 235–261.

[B40] Gross J. J. (1998). The emerging field of emotion regulation: an integrative review. *Rev. Gen. Psychol.* 2 271–299. 10.1037/1089-2680.2.3.271

[B41] Groth M. Grandey A. A. (2012). From bad to worse: negative exchange spirals in employee-customer service interactions. *Organ. Psychol. Rev.* 2 208–233. 10.1177/2041386612441735

[B42] Hadley C. N. (2014). Emotional roulette? symmetrical and asymmetrical emotion regulation outcomes from coworker interactions about positive and negative work events. *Hum. Relat.* 67 1073–1094. 10.1177/0018726714529316

[B43] Hakanen J. J. Perhoniemi R. Toppinen-Tanner S. (2008). Positive gain spirals at work: from job resources to work engagement, personal initiative and work-unit innovativeness. *J. Vocat. Behav.* 73 78–91. 10.1016/j.jvb.2008.01.003

[B44] Harris L. C. Ogbonna E. (2002). Exploring service sabotage: the antecedents, types and consequences of frontline, deviant, antiservice behaviors. *J. Serv. Res.* 4 163–183. 10.1177/1094670502004003001

[B45] Hayes A. F. (2013). *Introduction to Mediation, Moderation, and Conditional Process Analysis: A Regression-Based Approach*, Vol. 51. New York, NY: The Guilford Press, 335–337 10.1080/13557858.2017.1315056 28385036

[B46] Hobfoll S. E. (1989). Conservation of resources. A new attempt at conceptualizing stress. *Am. Psychol.* 44 513–524. 10.1037/0003-066X.44.3.513 2648906

[B47] Hobfoll S. E. (2001). The influence of culture, community, and the nested-self in the stress process: advancing conservation of resources theory. *Appl. Psychol.* 50 337–421. 10.1111/1464-0597.00062

[B48] Hüffmeier J. Hertel G. (2011). Many cheers make light the work: how social support triggers process gains in teams. *J. Manag. Psychol.* 26 185–204. 10.1108/02683941111112631

[B49] Hülsheger U. R. Lang J. W. Maier G. W. (2010). Emotional labor, strain, and performance: testing reciprocal relationships in a longitudinal panel study. *J. Occup. Health Psychol.* 15 505–521. 10.1037/a0021003 21058862

[B50] Hülsheger U. R. Schewe A. F. (2011). On the costs and benefits of emotional labor: a meta-analysis of three decades of research. *J. Occup. Health Psychol.* 16 361–389. 10.1037/a0022876 21728441

[B51] Jiang J. Y. Law K. S. Sun J. J. M. (2014). Leader-member relationship and burnout: the moderating role of leader integrity. *Manag. Organ. Rev.* 10 223–247. 10.1111/more.12022

[B52] Judge T. A. Woolf E. F. Hurst C. (2009). Is emotional labor more difficult for some than for others? a multilevel, experience-sampling study. *Pers. Psychol.* 62 57–88. 10.1111/j.1744-6570.2008.01129.x

[B53] Karasek R. A. Triantis K. P. Chaudhry S. S. (1982). Coworker and supervisor support as moderators of associations between task characteristics and mental strain. *J. Organ. Behav.* 3 181–200. 10.1002/job.4030030205

[B54] Kong H. Jeon J. E. (2018). Daily emotional labor, negative affect state, and emotional exhaustion: cross-level moderators of affective commitment. *Sustainability* 10 1–13. 10.3390/su10061967

[B55] Krannitz M. A. Grandey A. A. Liu S. Almeida D. A. (2015). Workplace surface acting and marital partner discontent: anxiety and exhaustion spillover mechanisms. *J. Occup. Health Psychol.* 20 314–325. 10.1037/a0038763 25705910 PMC4478215

[B56] Li J. Wong I. K. A. Kim W. G. (2017). Does mindfulness reduce emotional exhaustion? A multilevel analysis of emotional labor among casino employees. *Int. J. Hosp. Manag.* 64 21–30. 10.1016/j.ijhm.2017.03.008

[B57] Liden R. C. Wayne S. J. Meuser J. D. Hu J. Wu J. F. Liao C. W. (2015). Servant leadership: validation of a short form of the SL-28. *Leadersh. Q.* 26 254–269. 10.1016/j.leaqua.2014.12.002

[B58] Marcus B. Schuler H. (2004). Antecedents of counterproductive behavior at work: a general perspective. *J. Appl. Psychol.* 89 647–660. 10.1037/0021-9010.89.4.647 15327351

[B59] Maslach C. Schaufeli W. B. Leiter M. P. (2001). Job burnout. *Annu. Rev. Psychol.* 52 397–422. 10.1146/annurev.psych.52.1.397 11148311

[B60] Mayer D. M. Ehrhart M. G. Schneider B. (2009). Service attribute boundary conditions of the service climate-customer satisfaction link. *Acad. Manag. J.* 52 1034–1050. 10.5465/AMJ.2009.44635617

[B61] McCarthy J. M. Trougakos J. P. Cheng B. H. (2016). Are anxious workers less productive workers? It depends on the quality of social exchange. *J. Appl. Psychol.* 101:279. 10.1037/apl0000044 26375962

[B62] Mesmer-Magnus J. R. Dechurch L. A. Wax A. (2012). Moving emotional labor beyond surface and deep acting a discordance–congruence perspective. *Organ. Psychol. Rev.* 2 6–53. 10.1177/2041386611417746

[B63] Miles E. W. Patrick S. L. King W. C. Jr. (1996). Job level as a systemic variable in predicting the relationship between supervisory communication and job satisfaction. *J. Occup. Organ. Psychol.* 69 277–292. 10.1111/j.2044-8325.1996.tb00615.x

[B64] Monica M. Federica E. Margherita Z. Chiara G. Lara C. Cortese C. G. (2016). Inbound call centers and emotional dissonance in the job demands-resources model. *Front. Psychol.* 7:1133. 10.3389/fpsyg.2016.01133 27516752 PMC4964799

[B65] Muraven M. Tice D. M. Baumeister R. F. (1998). Self-control as limited resource: regulatory depletion patterns. *J. Pers. Soc. Psychol.* 74 774–789. 10.1037/0022-3514.74.3.774 9523419

[B66] Ng T. W. H. Sorensen K. L. (2008). Toward a further understanding of the relationships between perceptions of support and work attitudes: a meta-analysis. *Group Organ. Manag.* 33 243–268. 10.1177/1059601107313307

[B67] Parker S. K. Axtell C. M. (2001). Seeing another viewpoint: antecedents and outcomes of employee perspective taking. *Acad. Manag. J.* 44 1085–1100. 10.2307/3069390

[B68] Prati L. M. Liu Y. Perrewé P. L. Ferris G. R. (2009). Emotional intelligence as moderator of the surface acting—strain relationship. *J. Leadersh. Organ. Stud.* 15 368–380. 10.1177/1548051808328518 23244390

[B69] Ray E. B. Miller K. I. (1994). Social support, home/work stress, and burnout: who can help? *J. Appl. Behav. Sci.* 30 357–373. 10.1177/0021886394303007 15284625

[B70] Rosso B. D. Dekas K. H. Wrzesniewski A. (2010). On the meaning of work: a theoretical integration and review. *Res. Organ. Behav.* 30 91–127. 10.1016/j.riob.2010.09.001

[B71] Sakurai K. Jex S. M. (2012). Coworker incivility and incivility targets’ work effort and counterproductive work behaviors: the moderating role of supervisor social support. *J. Occup. Health Psychol.* 17 150–161. 10.1037/a0027350 22352293

[B72] Schaffer B. S. Riordan C. M. (2003). A review of cross-cultural methodologies for organizational research: a best- practices approach. *Organ. Res. Methods* 6 169–215. 10.1177/1094428103251542

[B73] Schaufeli W. B. Leiter M. P. Maslach C. Jackson S. E. (1996). “MBI-general survey,” in *Maslach Burnout Inventory Manual*, 3rd Edn, eds Maslach C. Jackson S. E. Leiter M. P. (Palo Alto, CA: Consulting Psychologists Press), 19–26.

[B74] Schmeichel B. J. Demaree H. A. Robinson J. L. Pu J. (2006). Ego depletion by response exaggeration. *J. Exp. Soc. Psychol.* 42 95–102. 10.1016/j.jesp.2005.02.005

[B75] Schneider B. Ehrhart M. G. Mayer D. M. Saltz J. L. Niles-Jolly K. (2005). Understanding organization-customer links in service settings. *Acad. Manag. J.* 48 1017–1032. 10.5465/AMJ.2005.19573107

[B76] Schneider K. T. Wesselmann E. D. Desouza E. R. (2017). Confronting subtle workplace mistreatment: the importance of leaders as allies. *Front. Psychol.* 8:1051–1064. 10.3389/fpsyg.2017.01051 28690576 PMC5481350

[B77] Shanock L. R. Allen J. A. Dunn A. M. Baran B. E. Scott C. W. Rogelberg S. G. (2013). Less acting, more doing: how surface acting relates to perceived meeting effectiveness and other employee outcomes. *J. Occup. Organ. Psychol.* 86 457–476. 10.1111/joop.12037

[B78] Sherony K. M. Green S. G. (2002). Coworker exchange: relationships between coworkers, leader-member exchange, and work attitudes. *J. Appl. Psychol.* 87 542–548. 10.1037/0021-9010.87.3.542 12090611

[B79] Skarlicki D. P. van Jaarsveld D. D. Shao R. Song Y. H. Wang M. (2016). Extending the multifoci perspective: The role of supervisor justice and moral identity in the relationship between customer justice and customer-directed sabotage. *J. Appl. Psychol.* 101 108–121. 10.1037/apl0000034 26052713

[B80] Skarlicki D. P. van Jaarsveld D. D. Walker D. D. (2008). Getting even for customer mistreatment: the role of moral identity in the relationship between customer interpersonal injustice and employee sabotage. *J. Appl. Psychol.* 93 1335–1347. 10.1037/a0012704 19025251

[B81] Sonnentag S. Fritz C. (2007). The recovery experience questionnaire: development and validation of a measure for assessing recuperation and unwinding from work. *J. Occup. Health Psychol.* 12 204–221. 10.1037/1076-8998.12.3.204 17638488

[B82] Solomon M. R. Surprenant C. Czepiel J. A. Gutman E. G. (1985). A role theory perspective on dyadic interactions: the service encounter. *J. Mark.* 49 99–111. 10.2307/1251180

[B83] Stucke T. S. Baumeister R. F. (2010). Ego depletion and aggressive behavior: is the inhibition of aggression a limited resource? *Eur. J. Soc. Psychol.* 36 1–13. 10.1002/ejsp.285

[B84] Thau S. Mitchell M. S. (2010). Self-gain or self-regulation impairment? Tests of competing explanations of the supervisor abuse and employee deviance relationship through perceptions of distributive justice. *J. Appl. Psychol.* 95 1009–1031. 10.1037/a0020540 20718511

[B97] The World Factbook (2017). *The World Factbook*. Available at: https://www.cia.gov/library/publications/resources/the-world-factbook/fields/2012.html

[B85] Thoits P. A. (2011). Mechanisms linking social ties and support to physical and mental health. *J. Health Soc. Behav.* 52 145–161. 10.1177/0022146510395592 21673143

[B86] Trougakos J. P. Beal D. J. Green S. G. Weiss H. M. (2008). Making the break count: an episodic examination of recovery activities, emotional experiences, and positive affective displays. *Acad. Manag. J.* 51 131–146. 10.5465/AMJ.2008.30764063

[B87] Viswesvaran C. Sanchez J. I. Fisher J. (1999). The role of social support in the process of work stress: a meta-analysis. *J. Vocat. Behav.* 54 314–334. 10.1006/jvbe.1998.1661 9325800

[B88] Wagner D. T. Barnes C. M. Scott B. A. (2014). Driving it home: How workplace emotional labor harms employee home life. *Pers. Psychol.* 67 487–516. 10.1111/peps.12044

[B89] Walsh G. Dahling J. J. Schaarschmidt M. Brach S. (2016). Surface-acting outcomes among service employees with two jobs. *J. Serv. Manag.* 27 534–562. 10.1108/JOSM-05-2015-0169

[B90] Wang K. L. Groth M. (2014). Buffering the negative effects of employee surface acting: the moderating role of employee–customer relationship strength and personalized services. *J. Appl. Psychol.* 99 341–350. 10.1037/a0034428 24079672

[B91] Wang M. Liao H. Zhan Y. Shi J. (2011). Daily customer mistreatment and employee sabotage against customers: examining emotion and resource perspectives. *Acad. Manag. J.* 54 312–334. 10.5465/AMJ.2011.60263093

[B92] Welsh D. T. Ellis A. P. Christian M. S. Mai K. M. (2014). Building a self-regulatory model of sleep deprivation and deception: the role of caffeine and social influence. *J. Appl. Psychol.* 99 1268–1277. 10.1037/a0036202 24611526

[B93] Wright T. A. Cropanzano R. (1998). Emotional exhaustion as a predictor of job performance and voluntary turnover. *J. Appl. Psychol.* 83 486–493. 10.1037/0021-9010.83.3.486 9648526

[B94] Wu L. Z. Tse E. C.-Y. Fu P. P. Hokwong K. Liu J. (2013). The impact of servant leadership on hotel employees’ “servant behavior”. *Cornell Hosp. Q.* 54 383–395. 10.1177/1938965513482519

[B95] Wu T. Y. Hu C. (2009). Abusive supervision and employee emotional exhaustion: dispositional antecedents and boundaries. *Soc. Sci. Electron. Pub.* 34 143–169. 10.1177/1059601108331217

[B96] Yagil D. Medlerliraz H. (2017). Personally committed to emotional labor: Surface acting, emotional exhaustion and performance among service employees with a strong need to belong. *J. Occup. Health Psychol.* 22 481–491. 10.1037/ocp0000049 27643607

